# Mitochondrial and Plastid Genomes of the Monoraphid Diatom *Schizostauron trachyderma*

**DOI:** 10.3390/ijms222011139

**Published:** 2021-10-15

**Authors:** Ewa Górecka, Romain Gastineau, Nikolai A. Davidovich, Olga I. Davidovich, Matt P. Ashworth, Jamal S. M. Sabir, Claude Lemieux, Monique Turmel, Andrzej Witkowski

**Affiliations:** 1 Institute of Marine and Environmental Sciences, University of Szczecin, Mickiewicza 16a, 70-383 Szczecin, Poland; andrzej.witkowski@usz.edu.pl; 2 Karadag Scientific Station—Natural Reserve of the Russian Academy of Sciences, p/o Kurortnoe, Feodosiya, 98188 Crimea, Russia; nickolaid@mail.ru (N.A.D.); olivdav@mail.ru (O.I.D.); 3 Department of Molecular Biosciences, NHB, University of Texas at Austin, Austin, TX 78712, USA; mashworth@utexas.edu; 4 Genomics and Biotechnology Research Group, Faculty of Science, King Abdulaziz University, Jeddah 21589, Saudi Arabia; jsabir@kau.edu.sa; 5 Département de Biochimie, Microbiologie et Bio-Informatique, Institut de Biologie Intégrative et des Systèmes, Université Laval, Québec, QC G1V 0A6, Canada; claude.lemieux@bcm.ulaval.ca (C.L.); monique.turmel@bcm.ulaval.ca (M.T.)

**Keywords:** monoraphid, biraphid, diatoms, organellar, genome, multigene, phylogeny, LAGLIDADG

## Abstract

We provide for the first time the complete plastid and mitochondrial genomes of a monoraphid diatom: *Schizostauron trachyderma.* The mitogenome is 41,957 bp in size and displays two group II introns in the *cox1* gene. The 187,029 bp plastid genome features the typical quadripartite architecture of diatom genomes. It contains a group II intron in the *petB* gene that overlaps the large single-copy and the inverted repeat region. There is also a group IB4 intron encoding a putative LAGLIDADG homing endonuclease in the *rnl* gene. The multigene phylogenies conducted provide more evidence of the proximity between *S. trachyderma* and fistula-bearing species of biraphid diatoms.

## 1. Introduction

The genus *Schizostauron* Grunow [1] represents one of the heterovalvar lineages of diatoms, where the two primary shells (valves) which make up the siliceous cell wall (frustule) have differing morphologies. In the case of *Schizostauron*, only one valve possesses raphe—longitudinal slits at the center of the valve which are involved in motility. Diatoms with this particular kind of heterovalvy are called “monoraphids”, and the two valves are labelled by their raphe (RV) or lack of raphe (SV). Taxa in *Schizostauron* can be distinguished from other monoraphid genera by the morphology of the transverse thickening of silica at the central area of the RV called a stauros. The genus is typical of temperate to tropical marine littoral zones. Species in this genus have often been misidentified as the monoraphid genera *Achnanthes* or *Cocconeis* and have a complicated taxonomic history, which includes the registration of invalid holotypes [1,2,3,4,5].

Since the mid-19th century, monoraphid diatoms have been classified in a separate evolutionary lineage featuring one raphe-loss event [6,7,8,9]. Treated as such, all described monoraphid genera were assigned to the order Achnanthales, including *Achnanthes* (Achnanthaceae), *Cocconeis*, *Campyloneis*, *Anorthoneis* (Cocconeidaceae), *Achnanthidium*, and *Eucocconeis* (Achnanthidiaceae). Round et al. [8] later split the genus *Achnanthes* sensu lato into several genera based on the re-evaluation of distinctive and shared morphological characters. The presence of a heterovalvate frustule is a shared feature of all members of *Achnanthes* sensu lato, but other morphological characters such as the girdle, orientation and shape of areola, and valve outline or central area structure are highly variable. The process of transferring taxa from *Achnanthes* and *Cocconeis* sensu lato into morphologically appropriate, newly established genera has continued over the last three decades and multiple new genera have been established [10,11,12,13,14,15,16], but despite these revisions, small groups of achnanthoid and cocconeid taxa remain without detailed generic accommodation.

Molecular phylogenetic studies of the raphid diatoms have supported the idea of multiple switches to the monoraphid state. These studies have shown *Schizostauron* to be monophyletic and closely related to other monoraphid genera such as *Astartiella*, *Madinithidium*, *Kolbesia*, and *Karayevia*. However, these “stauroneid” monoraphid genera (so named because of the presence of *Stauroneis* Ehrenberg sensu stricto in the molecular clade) are not monophyletic with respect to the other monoraphid genera of the Achnanthaceae, Achnanthidiaceae, and Cocconeidaceae, suggesting that their monoraphid states evolved independently [4,5,17].

Among other discriminating morphological characters, *Schizostauron* and monoraphid species included in the stauroneid have coaxial internal proximal raphe ends. This character is universal for the stauroneid genera *Stauroneis*, *Craticula* [8], and others that were recently studied using molecular data such as *Fistulifera*, *Proschkinia*, and *Sternimirus* [18,19,20,21]. In contrast, the genera in the Achnanthidiaceae and Cocconeidaceae feature proximal raphe ends bent internally into opposite directions [8,22]. The monoraphid species clearly need a revision of the higher-level taxonomy as they fail to form a natural group [22,23,24]. In addition, this could bring us closer to understanding the evolutionary origins of the different monoraphid genera, which would be the first step to elucidating the ecological or molecular selective pressures which lead to heterovalvy.

Here, we report the mitochondrial and plastid genome sequences of *Schizostauron trachyderma* (F.Meister) Górecka, Riaux-Gobin, and Witkowski, the first organellar genomes of a monoraphid diatom. These genomic data strengthen the phylogenetic position recently reported by Górecka et al. [5] and reveal that *Schizostauron* forms a clade within the Stauroneidaceae that is a sister to fistula-bearing *Fistulifera* and *Proschkinia* taxa. Several unusual features of the *S. trachyderma* organellar genomes will be discussed.

## 2. Results

### 2.1. Microscopy

**Figure 1:**
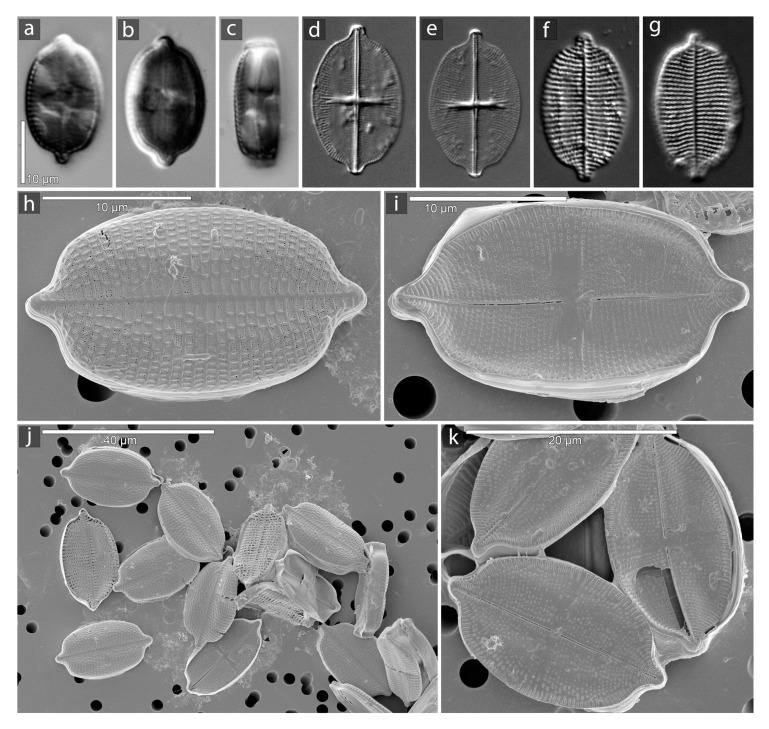
Light (LM) and Scanning electron microscopy (SEM) images of *S. trachyderma*. (**a**–**c**): live specimen with plastids. (**a**,**b**): valve view. (**c**): girdle view. (**d**–**g**): in LM. (**d**,**e**): raphe valve. (**f**–**g**): sternum valve. (**h**–**k**): in SEM. (**h**): sternum valve. (**i**): raphe valve.

Figure 1 illustrates the morphology of *S. trachyderma*, both in LM and SEM. The monoraphid morphology is apparent in comparing Figure 1d,e,i (RV) with Figure 1f–h (SV). The transverse stauros on the RV is particularly obvious in LM on Figure 1d,e.

### 2.2. Mitochondrial Genome

**Figure 2:**
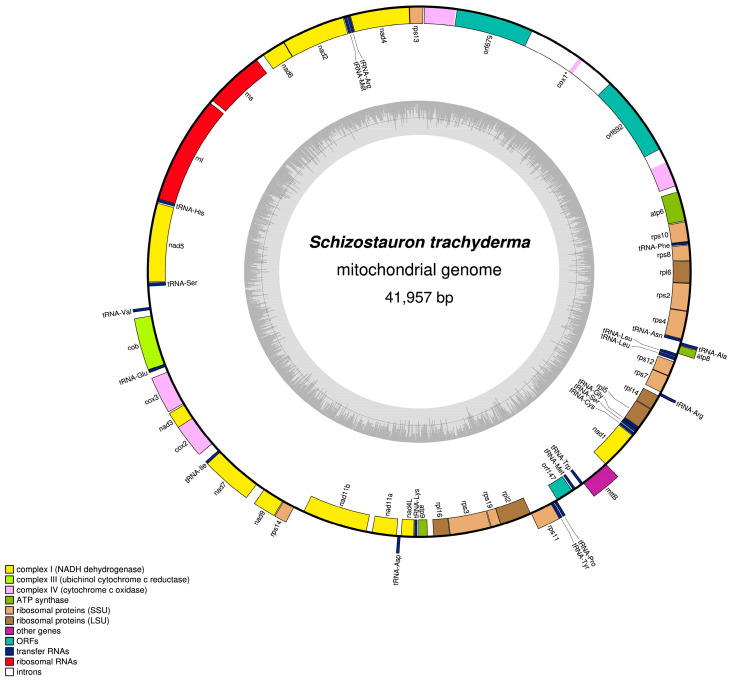
Map of the mitochondrial genome of *S. trachyderma*.

The 41,957 bp mitogenome of *S. trachyderma* (GenBank accession MZ520767) was retrieved with a coverage of 112X. It encodes the genes for 2 rRNAs, 22 tRNAs, and 35 proteins that include the two subunits of *Nad11* and the conserved orf147 [25] (Figure 2). Two group II introns of 3348 bp and 3325 bp in length interrupt *cox1* and both code for putative reverse transcriptases (orf692 and orf679, respectively). The *nad11-a* and *nad11-b* genes are adjacent to each other and are separated by only a 139-bp intergenic sequence. Note that the exact position of the start codon of *nad7* and *rpl6* remains ambiguous.

### 2.3. Comparative Analysis of Diatom Mitochondrial Genomes

**Figure 3:**
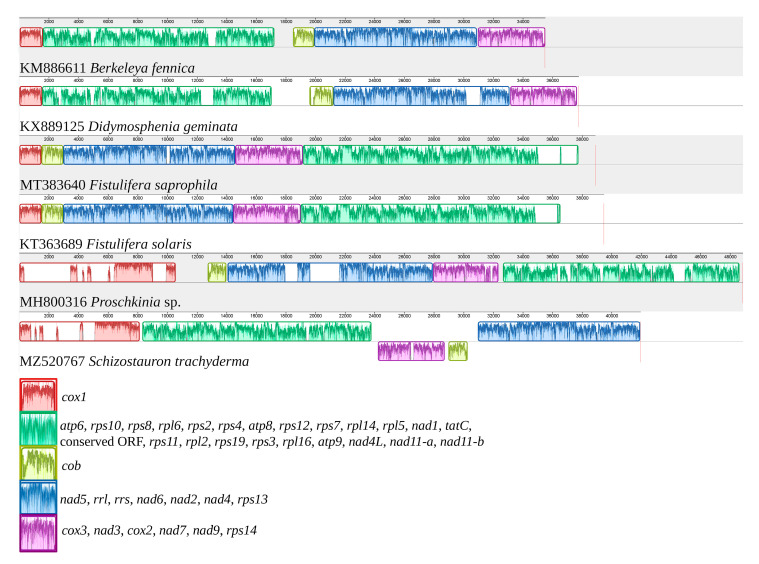
MAUVE alignment of the mitochondrial genomes of *Berkeleya fennica* (KM886611), *Didymosphenia geminata* (KX889125), *Fistulifera saprophila* (MT383640), *Fistulifera solaris* (KT363689), *Proschkinia* sp. (MH800316), and *Schizostauron trachyderma* (MZ520767). The legend below shows the gene content of the blocks of synteny (conserved protein-coding genes and rRNA only).

The mitochondrial genome of *S. trachyderma* was aligned to those of five closely related species identified in the phylogenetic analysis described below. In total, five blocks of synteny were detected in the MAUVE alignment (Figure 3). The mitogenome of *S. trachyderma* revealed a unique arrangement of these syntenic blocks, with the adjacent syntenic blocks formed by *cob* and by *cox3*, *nad3*, *cox2*, *nad7*, *nad9*, and *rps14* rearranged to the opposite DNA strand as compared to the other five genomes. Two sets of colinear mitochondrial genomes were identified: (1) those of *Berkeleya fennica* Juhlin-Dannfelt and *Didymosphenia geminata* (Lyngbye) M. Schmidt; and (2) those of *Fistulifera solaris* S. Mayama, M. Matsumoto, K. Nemoto, and T. Tanaka, as well as *Fistulifera saprophila* (Lange-Bertalot and Bonik) Lange-Bertalot and *Proschkinia* sp. These two sets of colinear mitogenomes differ only with respect to the position of a large syntenic block containing *atp6*, *rps10*, *rps8*, *rpl6*, *rps2*, *rps4*, *atp8*, *rps12*, *rps7*, *rpl14*, *rpl5*, *nad1*, *tatC*, *orf147*, *rps11*, *rpl2*, *rps19*, *rps3*, *rpl16*, *atp9*, *nad4L*, *nad11-a*, and *nad11-b*. The significantly larger mitogenome of *Proschkinia* sp. is mostly explained by the presence of introns in *cox1*.

### 2.4. Plastid Genome

**Figure 4:**
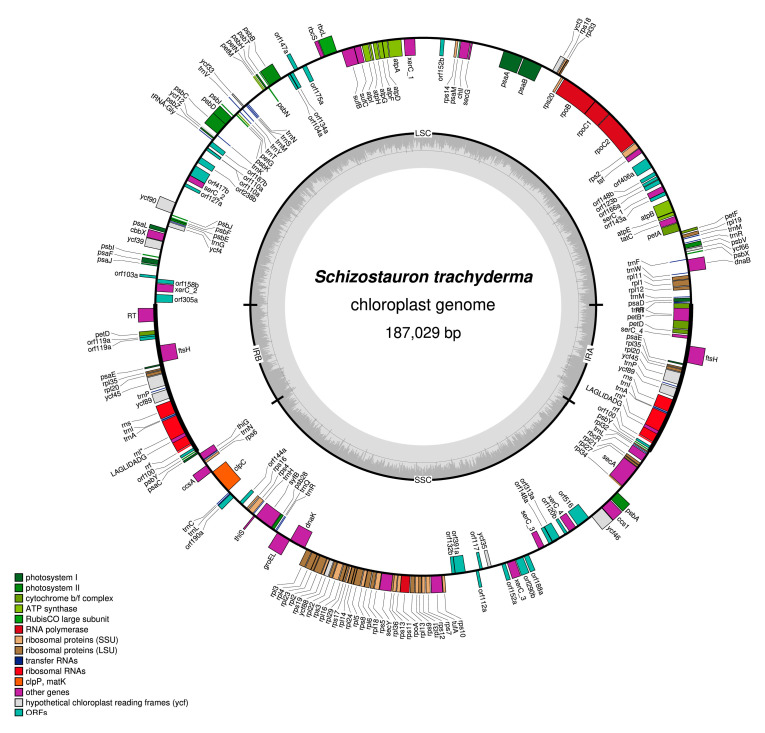
Map of the plastid genome of *S. trachyderma*.

The 187,029 bp plastid genome of *S. trachyderma* (GenBank accession MZ520768) exhibits the typical quadripartite architecture of diatom genomes (Figure 4). The large single-copy (LSC) region was retrieved with a coverage of 217X. It is 92,894 bp in size and encodes 67 conserved proteins, 15 tRNAs, and 22 open reading frames (ORFs) (Table 1). The closely linked orf104a and orf134a show similarities to *xerC* sequences coding for the putative integrases/recombinases. The putative protein of orf134a corresponds to the C-terminal domain of the integrases/recombinases and displays three of the conserved amino-acid residues present in this region (His-289, Arg-292, and Tyr-324), whereas the predicted protein of orf104a corresponds to the N-terminal domain of the integrases/recombinases but lacks the conserved Arg-173 typically present in this region. We speculate that these two ORFs are pseudogenes originating from a single large reading frame that was once coding for a functional protein. The predicted protein of orf110a shows some similarities with serine recombinases (*serC*), but its sequence is incomplete, lacking the C-terminal DNA binding site. The other putative *serC* (orf224a and orf227a) and *xerC* (orf299a and orf418a) proteins encoded by the plastid genome of *S. trachyderma* appear complete. In contrast, for example, to the *Haslea silbo* Gastineau, Hansen, and Mouget plastid genome that recently revealed all *serC* and *xerC* sequences in a single ca. 30 kb fragment located between *ycf35* and *psbA* [26], these elements are located in five distinct regions of the *S. trachyderma* (between the IR and *psaJ*, *ycf90* and *psbZ*, *psbB* and *rbcS*, *atpA* and *rps14*, *tsf* and *atpB*).

The small single-copy (SSC) region of *S. trachyderma* was retrieved with a coverage of 222X. It is 59,661 bp in size, and encodes 52 conserved proteins, 7 tRNAs, and 16 non-conserved ORFs, some of which encode putative *xerC* (orf294a and orf317a) and *serC* (orf234a) integrases/recombinases. The predicted protein of orf112a also shows some similarities with *xerC* integrases/recombinases but only retains the conserved Arg-173 and lacks the C-terminal region. Note that most of the ORFs encoding putative integrases/recombinases are located in a single block between *ycf46* and *rps10*.

The 17,237 bp inverted repeat (IR) region of the *S. trachyderma* plastid genome was retrieved with a coverage of 428X. It encodes nine proteins (*psbY*, *ycf89*, *ycf45*, *rpl20*, *rpl35*, *psaE*, *ftsH*, *petD*, *petB*), three rRNAs, three tRNAs, and three ORFs. The *petB* gene overlaps the IR and LSC regions and contains a 2287-bp group II intron coding for a putative reverse transcriptase (RT, orf494) that shows a high sequence similarity to the RTs identified in the same gene from the diatoms *Nanofrustulum shiloi* (J.J. Lee, Reimer, and McEnery) Round, Hallsteinsen and Paasche [27] and *Halamphora calidilacuna* J.G. Stepanek and Kociolek [28] (Table 1). Moreover, the introns of *S. trachyderma*, *N. shiloi*, and *H. calidilacuna* share the same insertion position between codons 7 and 8 of *petB*. Located between *ftsH* and *petB* of *S. trachyderma*, orf119 shows some similarities to *serC* sequences, but its N-terminal region is incomplete and lacks most of the presynaptic site 1 dimer interface.

**Figure 5:**
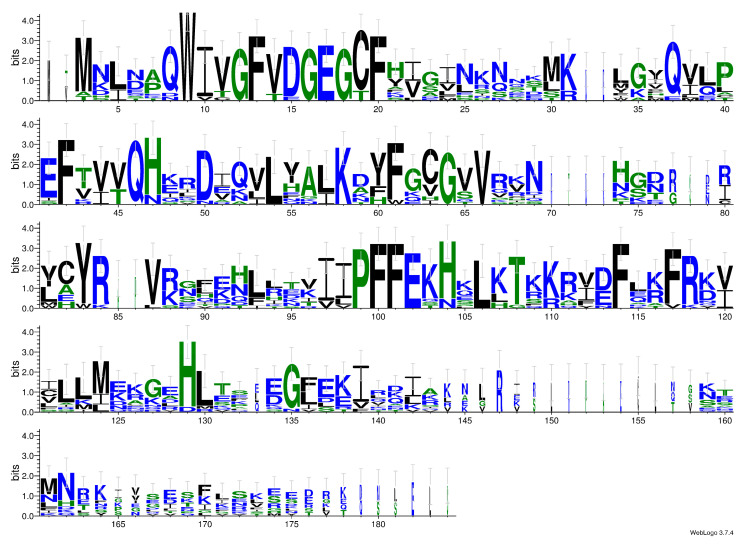
Sequence motifs obtained by aligning LAGLIDADG proteins from *Schizostauron trachyderma* and 13 sequences from various species of Chlorophyceae corresponding to IB4-L8 LAGLIDADG proteins. The alignment displayed as a LOGO shows the presence of two conserved motifs, QWIVGFVDG and PFFE.

The plastid gene for the large subunit ribosomal RNA (*rnl*) of *S. trachyderma* contains an IB4 intron of 657 bp that encodes a putative LAGLIDADG homing endonuclease (orf139). This intron is inserted between residues 1931 and 1932 relative to the 23S rRNA of *Escherichia coli* str. K-12, an insertion site that has been frequently observed among green algae [29,30]. The predicted protein of orf139 also shows strong similarity with the homing endonucleases identified in green algal IB4 introns at site 1931 [29]. As shown in the LOGO consensus of Figure 5, the typical QWIVGFVDG and PFFE motifs of LAGLIDADG homing endonucleases are highly conserved in the predicted protein of orf139.

### 2.5. Comparative Analyses of the Gene Content of Diatom Plastid Genomes

**Figure 6:**
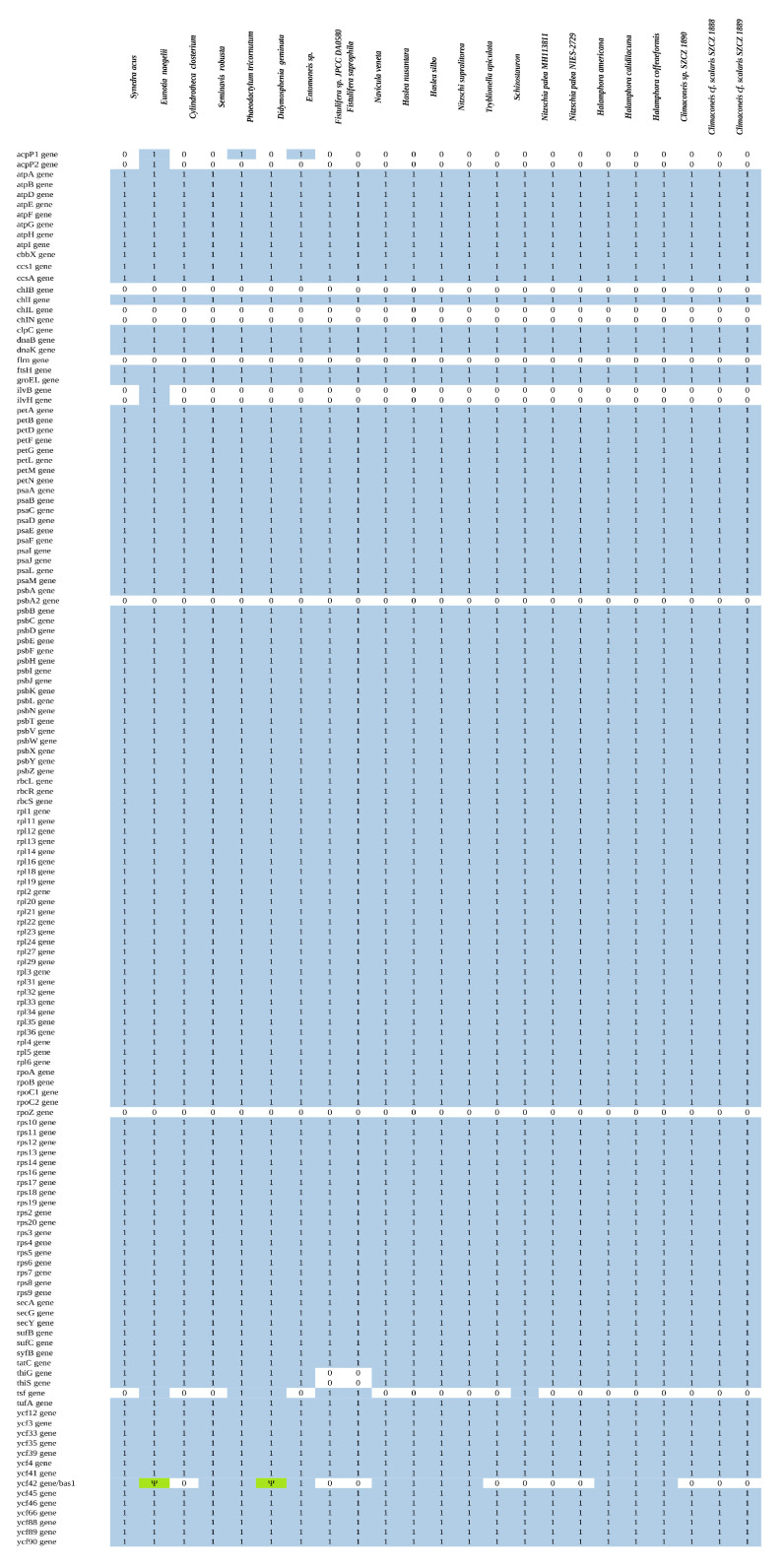
Comparison of the gene composition for the plastid genomes of all the species used in the phylogeny below. A 1/blue indicates the presence of the gene, a 0/white its lack, and a Ψ/green a pseudogene version of the gene.

The gene contents of plastid genomes from all available raphid diatoms were compared with that of *S. trachyderma*; a comparative table adapted from previous work [31] is displayed as Figure 6. The LSC contains a copy of the *tsf* gene, which codes for translation elongation factor T and often seems lost among raphid pennates, found only in *Fistulifera* spp., *D. geminata, Gomphoneis minuta* var. *cassiae* Kociolek and Stoermer, and *Phaeodactylum tricornutum* Bohlin. With the exception of *P. tricornutum*, all these species belong to the same clade in the plastid multigene phylogeny presented below, but this clade also contains *Climaconeis* spp., among which this gene was not found [32]. None of the Bacillariaceae (*Nitzschia* spp., *Tryblionella apiculata* W.Gregory) or Naviculaceae (*Haslea* spp., *Seminavis robusta* D.B. Danielidis and D.G.Mann, *Navicula veneta* Kützing) present it, nor do *Halamphora* spp. However, based on previous works [31], this gene is also absent from almost three-quarters of the plastid genomes sequenced, including most centric and araphid pennate species. *Schizostauron trachyderma* possesses the two genes *thiS* and *thiG*, shared by all the other species of the cluster, except *Fistulifera* spp. *Schizostauron trachyderma*, which is missing the *bas1*/*ycf42* gene. This gene is also missing among most Bacillariaceae and Naviculaceae but has been found in *Nitzschia supralitorea* Lange-Bertalot. Among the species clustered by phylogeny, only *D. geminata* presents a pseudogene version of *bas1*/*ycf42*.

### 2.6. Multigene Phylogenies

**Figure 7:**
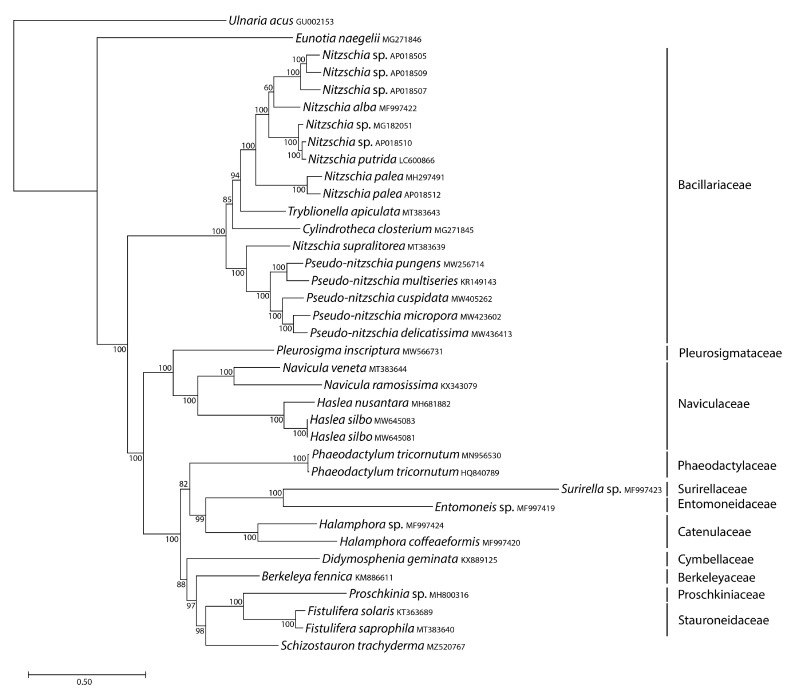
Maximum likelihood phylogeny inferred from an alignment of concatenated protein-coding genes from 37 mitochondrial genomes of diatoms, including that of *S. trachyderma*. The araphid species *Ulnaria acus* served as an outgroup in this analysis. The best scoring RAxML tree (log likelihood = −414,469.369802) is presented with bootstrap support values denoted on the nodes. The scale bar indicates the number of substitutions per site. The araphid species *Ulnaria acus* is used as an outgroup.

**Figure 8:**
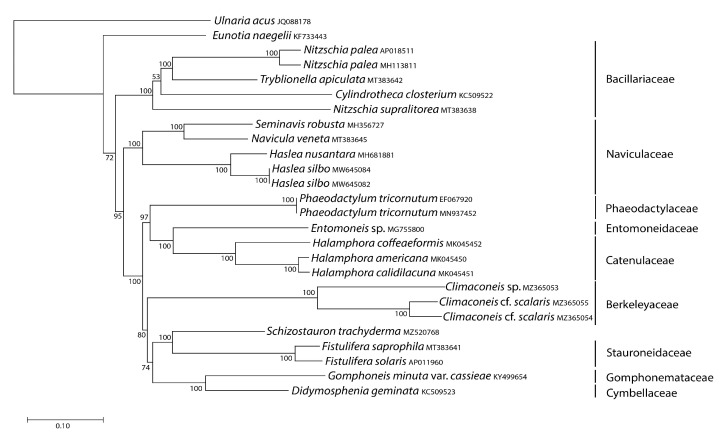
Maximum likelihood phylogeny inferred from an alignment of concatenated protein-coding genes from 26 plastid genomes of diatoms, including that of *S. trachyderma*. The araphid species *Ulnaria acus* served as an outgroup in this analysis. The best scoring RAxML tree (log likelihood = −878,702.878358) is presented with bootstrap support values denoted on the nodes. The scale bar indicates the number of substitutions per site.

We inferred mitochondrial and plastid phylogenomic trees from the concatenated gene sequences of *S. trachyderma* and all raphid pennate diatom organelle genome sequences available in GenBank, using an araphid pennate species as an outgroup. *Schizostauron trachyderma* proved to be a sister to *Fistulifera* spp. (and *Proschkinia* sp. in the case of the mitochondrial data) in all trees (Figure 7 and Figure 8), a result consistent with recently reported phylogenetic analyses [5,20,21]. Note that *Fistulifera* and *Proschkinia* taxa present a distinctive occluded pore on their valves, called a fistula. Interestingly, this feature is not found on *Schizostauron* valves.

The mitogenome-based tree (Figure 7) is the most taxon-rich of both trees, mostly because of the availability of several mitogenomes from two genera of the Bacillariaceae (*Nitzschia* and *Pseudo-nitzschia*). In addition to the *Schizostauron, Fistulifera,* and *Proschkinia* association mentioned above, the mitochondrial tree revealed that *S. trachyderma* was part of a larger clade including *Surirella* sp., *Halamphora* spp., *Entomoneis* sp., *P. tricornutum*, *D. geminata*, and *B. fennica*. The Naviculaceae formed a monophyletic group sister to a clade containing the rest of the raphid pennates.

Although the plastome-based phylogenetic tree (Figure 8) has a smaller taxon sampling, its topology is similar to the mitochondrial tree. The *S. trachyderma* also proved to be a sister of *Fistulifera* spp. and these taxa were recovered in a clade that also includes *G. minuta* var. *cassiae*, *D. geminata*, and *Climaconeis* spp.

## 3. Discussion

The multigene phylogenetic analyses presented here confirm the close evolutionary relationship between *S. trachyderma* and fistula-bearing taxa such as *Fistulifera* spp. and *Proschkinia* sp. This phylogenetic association could be improved and refined in future studies of additional organellar genomes of monoraphid genera such as *Astartiella, Madinithidium*, *Karayevia*, and *Kolbesia.* Based on recently published analyses, we expect these genera to be related to *Schizostauron,* as these stauroneid monoraphid taxa form a monophyletic clade sister to *Fistulifera* and *Proschkinia*, which is nested in a clade comprising genera belonging to Stauroneidaceae, such as *Stauroneis*, *Craticula*, *Sternimirus*, *Dorofeyukea*, *Parlibellus*, and *Prestauroneis* [5,17,18,20,21]. It is possible that the close relationship of stauroneid biraphid genera to stauroneid monoraphid genera is a result of insufficient taxon sampling or inadequate or insufficient choice of molecular markers. Genomic data from these taxa might reveal signature characters of specific monoraphid clades and could provide insights to the independent losses of the raphe on one valve.

The *S. trachyderma* plastid genome revealed features that were rarely or never observed among diatoms. The finding of a group II intron overlapping the IR and the LSC regions in the *petB* gene is certainly noteworthy, as is the observation of a LAGLIDADG homing endonuclease in the *rnl* gene. To our knowledge, genes encoding this type of homing endonuclease have so far been found only in the IA3 *rnl* intron of the *S. robusta* plastid genome (annotated as I-SroI, accession AZJ16657.1 [33] and in a *cox1* intron of the *N. supralitorea* mitogenome, accession QWM93242.1 [34]. As in a few other diatom species [31], our analyses of conserved domains in the putative *serC* and *xerC* genes of *S. trachyderma* suggest that some of them are pseudogenes. As interesting as both the above-mentioned results are for the study of mobile DNA, we refrain from speculating on any explanation regarding their presence/absence among different taxa.

Our study, the first of its kind on a monoraphid diatom, should soon be followed by more organellar genome sequencing on other species belonging to genera such as the aforementioned *Parlibellus*, *Stauroneis*, *Astartiella*, and *Madinithidium.*

## 4. Materials and Methods

### 4.1. Isolation and Cultivation of the Biological Material

The strain SZCZE1420 of *S. trachyderma* was isolated from an environmental sample collected in February 2015 near Jeddah on the Red Sea coast of Saudi Arabia (21.7561° N 39.05° E). A monoclonal culture was established using glass micropipettes and inverted light microscopy (Nikon eclipse TS100) following Andersen and Kawachi [35]. The culture is kept in artificial f/2 culture medium [36] in a growth chamber (Biogenet, Poland) with 12 h day (20 °C):12 h night (18 °C) cycles under 100 μmol photons m^−2^ s^−1^ illumination.

### 4.2. Light and Scanning Electron Microscopy

Diatom pellets were centrifuged at 900× *g* for 5 min and treated with 37% hydrogen peroxide for 3 h at 170 °C to remove organic components of frustules. The residual material was washed 7–10 times with distilled water. For light microscopy (LM), cleaned frustules were pipetted onto coverslips, air-dried, and mounted on glass slides with synthetic diatom resin Naphrax^®^ (Brunel Microscopes Ltd., Chippenham, UK). LM microphotographs of cleaned frustules and plastids were taken at the University of Szczecin by means of a Zeiss Axio Scope A1 (Carl Zeiss, Jena, Germany) with an oil immersion lens Zeiss Plan-Apochromat 100×/1.40 Oil M27 (Carl Zeiss, Jena, Germany) using a Canon EOS 500D camera (Canon, Tokyo, Japan) with the Canon EOS Utility software. For scanning electron microscopy (SEM), the cleaned material was pipetted onto a Whatman Nuclepore polycarbonate membrane filter with 5 µm pores (cat. no. 110613, Maidstone, UK) and mounted onto aluminium stubs. SEM observations were performed using a Hitachi SU8010 (Tokyo, Japan) at the University of Rzeszów (Poland), Faculty of Agriculture and Biology.

### 4.3. DNA Sequencing, Annotation, Whole Genome Alignments and Phylogeny

The SZCZE1420 clone of *S. trachyderma* was grown as described above, and cells in the exponential growth phase were harvested by centrifugation. DNA was extracted according to Doyle and Doyle [37]. Sequencing took place on the DNBseq platform at the Beijing Genomics Institute (Shenzhen). A total of 40 million 150-bp paired-end reads were assembled using SPAdes 3.14.0 [38] with a k-mer of 125. Contigs were verified and merged using Consed [39]. The genes were identified as previously described [34,40]. Maps of organellar genomes were prepared using the OGDRAW v1.3.1 online platform [41]. Whole mitogenome alignment was performed with progressive Mauve [42] after removing the second copy of the IR sequence in the plastid genomes. LOGO consensus sequences of LAGLIDADG homing endonucleases were obtained online with WebLogo3 [43]. For multigene phylogenies, mitochondrial and plastid protein-coding genes were extracted, separately concatenated, and aligned with orthologous diatom gene sequences obtained from GenBank. The mitochondrial and plastid multigene phylogeny were conducted on 37 and 26 taxa, respectively, including *S. trachyderma* and *U. acus* in both cases. The sampling was restricted to raphid pennate species, except for the araphid species *Ulnaria acus* (Kützing) Aboal that served as an outgroup. Phylogenetic analyses were conducted using RaxML version 8 [44], with the GTR + I + G model and 1000 bootstrap replications. Genes were concatenated and aligned using MAFFT 7 [45] before the alignments were trimmed by trimAl v1.2 [46]. The evolution model was chosen using jModelTest2 v2.1.10 [47] on the trimmed alignments. The number of concatenated genes was 35 for the mitochondrial-inferred phylogeny and 127 for the plastid-inferred phylogeny. The final sizes of the alignments as calculated by trimAl were 22140 bp for the mitochondrial genes alignment and 86587 bp for the plastid genes alignment. The evolution model was used as a single model across the entire alignment.

## Figures and Tables

**Table 1:** List of the various ORF found in the mitochondrial and plastid genomes of *Schizostauron trachyderma*, with the name, position, putative conserved domains, and best blastp result.

Name	Position	Putative Conserved Domain	Best Blastp Result (E-Value, Identity)
orf143a	Plastid genome (LSC)	None	CAA45586.1 from *Cylindrotheca closterium* (2 × 10^−58^, 69.23%)
orf227a	Plastid genome (LSC)	Putative serine recombinase	AZJ16760.1 from *Semiavis robusta* (9 × 10^−128^, 85.31%)
orf166a	Plastid genome (LSC)	None	AZJ16664.1 from *Seminavis robusta* (2 × 10^−24^, 36.88%)
orf123b	Plastid genome (LSC)	None	WP_101018572.1 from *Olleya* sp. (3 × 10^−6^, 34.29%)
orf148b	Plastid genome (LSC)	None	AXF37983.1 from *Seminavis robusta* (6 × 10^−35^, 66.41%)
orf406a	Plastid genome (LSC)	None	CAA45586.1 from *Cylindrotheca closterium* (0.0, 91.75%)
orf152b	Plastid genome (LSC)	None	YP_009028997.1 from *Cylindrotheca closterium* (2 × 10^−33^, 44.30%)
orf418a	Plastid genome (LSC)	Putative integrase recombinase	YP_009686230.1 from *Halamphora calidilacuna* (1 × 10^−39^, 50.00%)
orf175a	Plastid genome (LSC)	None	QUW40432.1 from *Haslea silbo* (5 × 10^−21^, 33.88%)
orf147a	Plastid genome (LSC)	None	None significant
orf134a	Plastid genome (LSC)	Putative integrase recombinase (not complete, contains His-289, Arg-292, and Tyr-324)	YP_009029005.1 from *Cylindrotheca closterium* (5 × 10^−63^, 74.63%)
orf104a	Plastid genome (LSC)	Putative integrase recombinase (not complete)	YP_009686252.1 from *Halamphora calidilacuna* (2 × 10^−27^, 56.31%)
orf187b	Plastid genome (LSC)	None	HAC63963.1 from *Cyanothece* sp. (1 × 10^−119^, 94.59%)
orf110a	Plastid genome (LSC)	Putative serine recombinase (not complete in C terminal, lacks the DNA binding site)	QGW12742.1 from *Nanofrustulum shiloi* (1 × 10^−47^, 85.26%)
orf238b	Plastid genome (LSC)	None	QUS63763.1 from *Haslea silbo* (3 × 10^−23^, 35.02%)
orf417b	Plastid genome (LSC)	None	AXF37982.1 from *Seminavis robusta* (5 × 10^−53^, 30.82%)
orf224a	Plastid genome (LSC)	Putative serine recombinase	YP_009496149.1 from *Plagiogrammopsis vanheurckii* (6 × 10^−135^, 88.79%)
orf127a	Plastid genome (LSC)	None	None significant
orf103a	Plastid genome (LSC)	None	YP_009028999.1 from *Cylindrotheca closterium* (6 × 10^−17^, 45.79%)
orf158b	Plastid genome (LSC)	None	YP_009029000.1 from *Cylindrotheca closterium* (4 × 10^−25^, 52.94%)
orf299a	Plastid genome (LSC)	Putative integrase recombinase	YP_009497021.1 from *Psammoneis obaidii* (7 × 10^−151^, 76.77%)
orf305a	Plastid genome (LSC)	None	AZJ16668.1 from *Seminavis robusta* (2 × 10^−154^, 74.43%)
orf494a	Plastid genome (IR)	Putative reverse transcriptase	QGW12739.1 from *Nanofrustulum shiloi* (0.0, 58.17%)
orf119	Plastid genome (IR)	Putative serine recombinase (not complete in N terminal, lacks most of the presynaptic site 1 dimer interface)	QGW12742.1 from *Nanofrustulum shiloi* (6 × 10^−56^, 68.03%)
orf139	Plastid genome (IR)	Putative LAGLIDADG	AAL34315.1 from *Pterosperma cristatum* (2 × 10^−66^, 77.34%)
orf100	Plastid genome (IR)	None	AZJ16668.1 from *Seminavis robusta* (5 × 10^−9^, 68.29%)
orf190a	Plastid genome (SSC)	None	YP_009308934.1 from *Toxarium undulatum* (2 × 10^−65^, 61.96%)
orf144a	Plastid genome (SSC)	None	None significant
orf132b	Plastid genome (SSC)	None	AZJ16659.1 from *Seminavis robusta* (2 × 10^−71^, 90.52%)
orf391a	Plastid genome (SSC)	None	CAA45582.1 from *Cylindrotheca fusiformis* (2 × 10^−168^, 73.63%)
orf112a	Plastid genome (SSC)	Putative integrase recombinase (not complete in C terminal, displays only the first conserved Arg-173)	YP_009028833.1 from *Asterionellopsis glacialis* (1 × 10^−47^, 90.00%)
orf117c	Plastid genome (SSC)	None	CAA45586.1 from *Cylindrotheca closterium* (2 × 10^−50^, 74.77%)
orf152a	Plastid genome (SSC)	None	HAC63961.1 from *Cyanothece* sp. (3 × 10^−102^, 97.32%)
orf317a	Plastid genome (SSC)	Putative integrase recombinase	NNF85095.1 from *Winogradskyella* sp. (4 × 10^−88^, 53.01%)
orf290b	Plastid genome (SSC)	None	YP_009028832.1 from *Asterionellopsis glacialis* (8 × 10^−61^, 51.33%)
orf188a	Plastid genome (SSC)	None	AZJ16664.1 from *Seminavis robusta* (3 × 10^−93^, 76.63%)
orf234a	Plastid genome (SSC)	Putative serine recombinase	QWM93463.1 from *Tryblionella apiculata* (1 × 10^−140^, 88.53%)
orf148a	Plastid genome (SSC)	None	YP_009059189.1 from *Eunotia naegelii* (3 × 10^−15^, 32.89%)
orf313a	Plastid genome (SSC)	None	QUS63950.1 from *Haslea silbo* (5 × 10^−28^, 51.55%)
orf120b	Plastid genome (SSC)	None	None significant
orf294a	Plastid genome (SSC)	Putative integrase recombinase	YP_009495909.1 from *Plagiogramma staurophorum* (6 × 10^−176^, 83.33%)
orf516a	Plastid genome (SSC)	None	CAA45586.1 from *Cylindrotheca closterium* (2 × 10^−92^, 34.58%)
orf645	Mitogenome	Putative reverse transcriptase	YP_009317775.1 from *Navicula ramosissima* (0.0, 82.20%)
orf690	Mitogenome	Putative reverse transcriptase	QUS63794.1 from *Haslea silbo* (0.0, 93.14%)

## Data Availability

Molecular data have all been deposited to GenBank. Data are also available on Zenodo with the following link: https://doi.org/10.5281/zenodo.5361687 (accessed on 1 September 2021).
